# Dynamic reverse phase transformation induced high-strain-rate superplasticity in low carbon low alloy steels with commercial potential

**DOI:** 10.1038/s41598-017-09493-7

**Published:** 2017-08-23

**Authors:** Wenquan Cao, Chongxiang Huang, Chang Wang, Han Dong, Yuqing Weng

**Affiliations:** 1 Special Steel department of Central Iron and Steel Research Institute (CISRI), Beijing, 100081 China; 2 School of Aeronautics and Astronautics, Sichuan University, Chengdu, 610065 China

## Abstract

Superplastic materials are capable of exhibiting large tensile elongation at elevated temperature, which is of great industrial significance because it forms the basis of a fabrication method to produce complex shapes. Superplasticity with elongation larger than 500% has been widely realized in many metals and alloys, but seldomly been succeeded in low carbon low alloy steel, even though it is commercially applied in the largest quantity. Here we report ultrahigh superplastic elongation of 900–1200% in the FeMnAl low carbon steels at high strain rate of 10^−2^–10^−3^ s^−1^. Such high-strain-rate superplasticity was attributed to dynamic austenite reverse phase transformation from a heavily cold rolled ferrite to fine-grained ferrite/austenite duplex microstructure and subsequent limited dynamic grain coarsening, under which a large fraction of high angle boundaries can be resulted for superplastic deformation. It is believed that this finding of the low carbon low alloy steel with ultrahigh superplasticity and relative low cost would remarkably promote the application of superplastic forming technique in automobile, aeronautical, astronautical and other fields.

## Introduction

Superplasticity is referred to the ability of a material to sustain large plastic deformation (elongation-to-failure: ΔL/L_0_ > 100%) in tension. Since the pioneering finding of superplasticity by Rosenhain^1^, extensive efforts have been made over the past century, and now the superplasticity with ΔL/L_0_ larger than 500% can be realized readily in a wide range of alloy systems^2^, such as titanium alloys^3, 4^, aluminum alloys^5, 6^, and magnesium alloys^7^. A number of steels and ferrous alloys exhibit superplasticity as well but usually at very low strain rate^8^. For example, the ultrahigh carbon steels and duplex stainless steels show very high superplasticity (ΔL/L_0_ > 1000%) at both low strain rate of 10^−4^ s^−1^ and high temperature^8–11^. As to low carbon low alloy steels, since the fist work by Morrison in 1968^12^ the highest superplasticity of 800% in a 0.2 wt.%C steel was obtained only at a very low strain rate of 1.67 × 10^−4^ s^−1^ during the past half century^2, 8, 13^. Therefore, it has been commonly accepted that low carbon low alloy steels are not superplastic materials although they are commercially produced and applied in the largest quantity in industry. On the other hand, superplastic forming is an attractive option for components with complex shapes to be formed at lower stress and lower tooling cost than that of conventional cold pressing forming. Due to the low density and high superplasticity, aluminum alloys and titanium alloys were succeeded in fabricating automotive and airplane components by means of superplastic forming and diffusion bonding^13^. However, the superplastic forming of aluminum and titanium were usually expensive, which is about 5 and 20 times higher than the cost of commonly applied steels. Therefore, developing superplastic low carbon steels with high-strain-rate superplasticity, high strength, light weight and low cost are urgently needed to face the requirements from natural energy limitation, environmental pollution and stringent safety law in automobile, aeronautical, astronautical industry.

Here, we demonstrate newly designed low carbon low alloy steels with excellent high-strain-rate superplasticity. This new designed steel was initially developed as the 3^rd^ generation automobile steel with a product of strength-ductility of about 30 GPa% by Central Iron and Steel Research Institute of China (CISRI)^14, 15^, which was shown to be economical and feasible to produce lightweight and complex shape components for today’s automobiles. The chemical composition is given in Extended Data Table 1. The cast ingots of about 50 kg were produced by conventional melting and mold casting. Then these ingots were forged and hot rolled into sheets in thickness of about 6 mm and air cooled to room temperature (RT), thereafter softened at 650 °C and further RT coldly rolled to 1.8 mm in thickness. Tensile samples having gauge length of 5 mm, width of 8 mm and thickness of 1.8 mm were cut from the cold rolled coils. The tensile experiments were carried out at an initial strain rate of 10^−3^–10^−1^ s^−1^ on MTS880 testing machine equipped with a heating furnace without atmosphere protection. Before tensile test, the specimens were heated to a given temperature with heating rate of about 2 °C s^−1^ and holding for 5 minutes to get a uniform temperature distribution.

**Figure 1:**
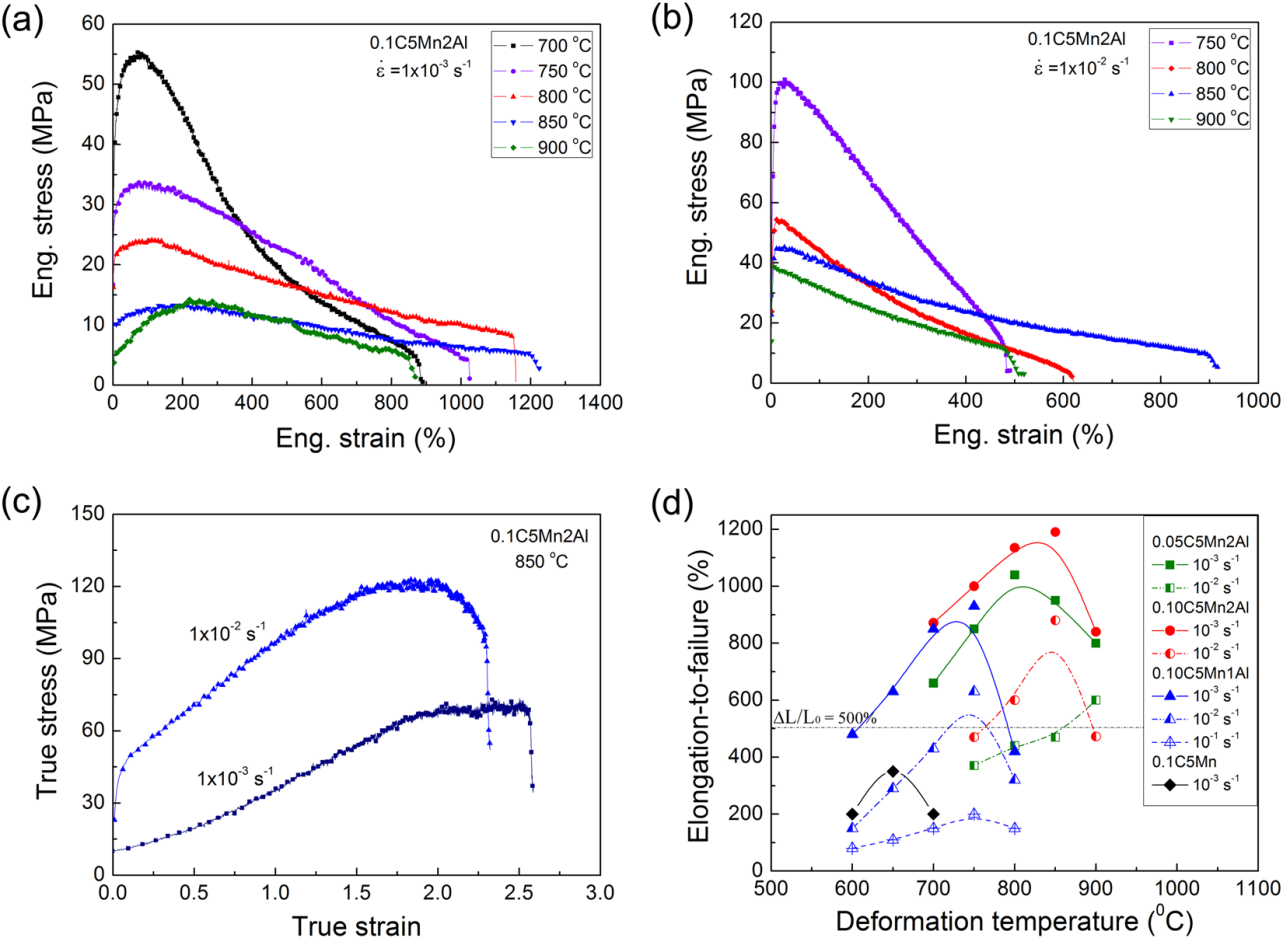
Tensile superplasticity of FeMnAl low carbon steels obtained at different initial strain rates and temperatures. (**a**) Engineering stress-strain curves of 0.10C5Mn2Al steel at 1 × 10^−3^ s^−1^. (**b**) Engineering stress-strain curves of 0.10C5Mn2Al steel at 1 × 10^−2^ s^−1^. (**c**) Representative true stress-strain curves converted from the curves in (**a**) and (**b**) using standard formula to demonstrate strain hardening. (**d**) Plot of elongation-to-failure against temperature.

The engineering stress-strain curves of the studied steels are presented in Fig. 1a and b, as well as in Extended Data Figs 1 and 2. Generally, the lower the strain rate and the higher the temperature, the lower are the yield and flow stress. It is interesting that strong strain hardening is identified as well (Fig. 1c and Extended Data Figs 1 and 2), which is useful to impede strain localization (e.g. necking) and improve uniform plastic deformation in metals and alloys^16^. The relationship between ΔL/L_0_ and deformation temperature is plotted in Fig. 1d. It shows that in a wide temperature range of 600–900 °C and at strain rate of 1 × 10^−3^ s^−1^, the steels can meet the critical value (ΔL/L_0_ > 500%) required for superplastic forming in industry. An optimal temperature exhibiting the highest superplasticity can be indentified based on Fig. 1d.

For instance, the 0.10C5Mn2Al steel has ΔL/L_0_ of 1190% at 850 °C, the 0.10C5Mn1Al steel has ΔL/L_0_ of 930% at 750 °C and the 0.05C5Mn2Al steel has ΔL/L_0_ of 1040% at 800 °C. Particularly, the 0.10C5Mn2Al steel shows excellent superplasticity (ΔL/L_0_ = 880%) at a high strain rate of 1 × 10^−2^ s^−1^ (Fig. 1b and c), which is industrially significant for rapid superplastic forming. The samples before and after deformation are shown in Extended Data Fig. 3, which demonstrates the excellent superplastic deformation capability of the studied steels.

**Figure 2:**
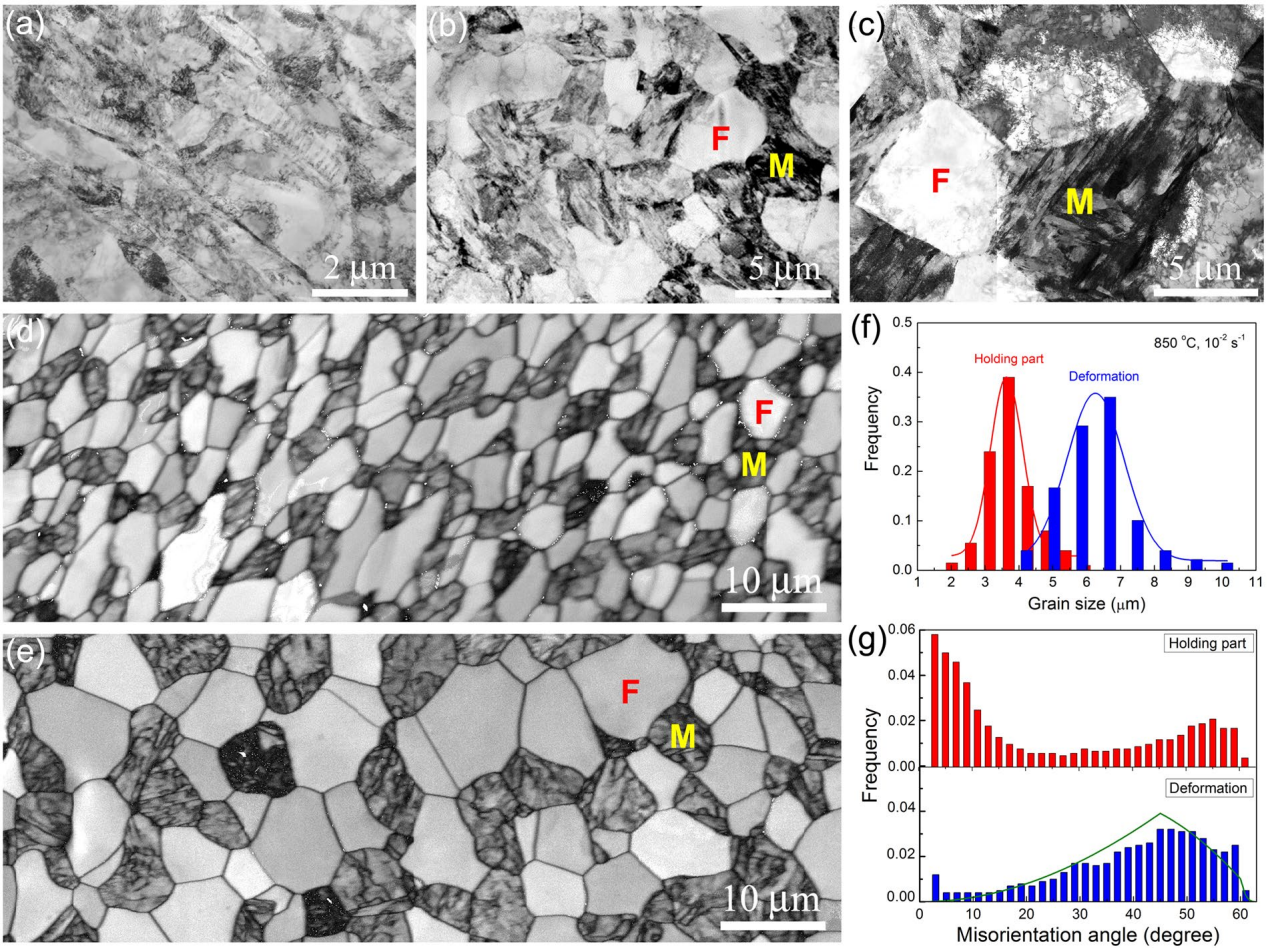
Microstructural characteristics of the studied 0.10C5Mn2Al steel before and after superplastic deformation at 850 °C and 1 × 10^−2^ s^−1^. (**a**) TEM microstructure of the starting cold rolled sample. (**b**) TEM microstructure in the holding portion of tensile sample. (**c**) TEM microstructure in the deformed gauge section of tensile sample. (**d**) EBSD gray image showing microstructure in the holding portion. (**e**) EBSD gray image showing microstructure in the deformed gauge section. In (**b–d**), the bright clean grains are ferrite (F), while the dark grains with complex contrast are martensite (M) formed by phase transformation from austenite during air cooling. (**f**) Grain size distributions in the holding portion and deformed gauge section. The grain size is defined with high-angle GB (>15°). (**g**) Misorientation distributions in the holding portion and deformed gauge section.

It is widely accepted that the dominant mechanism for structural superplasticity is boundary sliding (both grain boundary (GB) and phase boundary), which is usually accompanied with grain rotation and grain growth^2, 17^. Therefore, enhancing the fraction of high-angle GBs (>15°) is considered as the primary route to improve superplasticity in metals and alloys. Accordingly, refining grain size was used to fulfill this requirement and thus achieve high-strain-rate and low-temperature superplasticity in nanostructured materials fabricated by severe plastic deformation or electrodeposition^6, 7, 18^. In this study, TEM observation in Fig. 2a shows that the starting sample is a typical cold rolled ferrite with low fraction of high angle GBs, which is obviously different from common superplastic materials with initial fine grain size and largely fractioned high angle GBs^4–10^. When deforming the sample at elevated temperature dynamic reverse phase transformation from ferrite to austenite took place. Figure 2b–e reveal the formation of fine martensite grains in the sample deformed at 1 × 10^−2^ s^−1^ and at 850 °C. The volume fraction of marteniste is measured to be about 56 ± 4% and 52 ± 3% in deformed section and holding portion, respectively. These martensite grains originated from austenite by phase transformation during air cooling after superplastic deformation. As a result, a number of high-angle GBs were produced among the fine equiaxed grains in both ferrite and austenite, which satisfied the precondition of superplastic deformation. At the same time, the increase of dynamic grain growth and high-angle GBs occurred as well with increasing deformation. As shown in Fig. 2f, in comparison with the grain size of 3.6 ± 0.5 μm in the holding portion without deformation the average grain size in the deformed gauge section grew up to 6.2 ± 0.4 μm. The limited grain growth increases the fraction of high-angle GBs significantly from 51.2 ± 3% in the holding portion to 90.4 ± 2% in the deformed gauge section, as seen in Fig. 2g. The misorientation distribution is very close to the random orientation distribution, implying that strong GB sliding and grain rotation took place during superplasticity.

**Figure 3:**
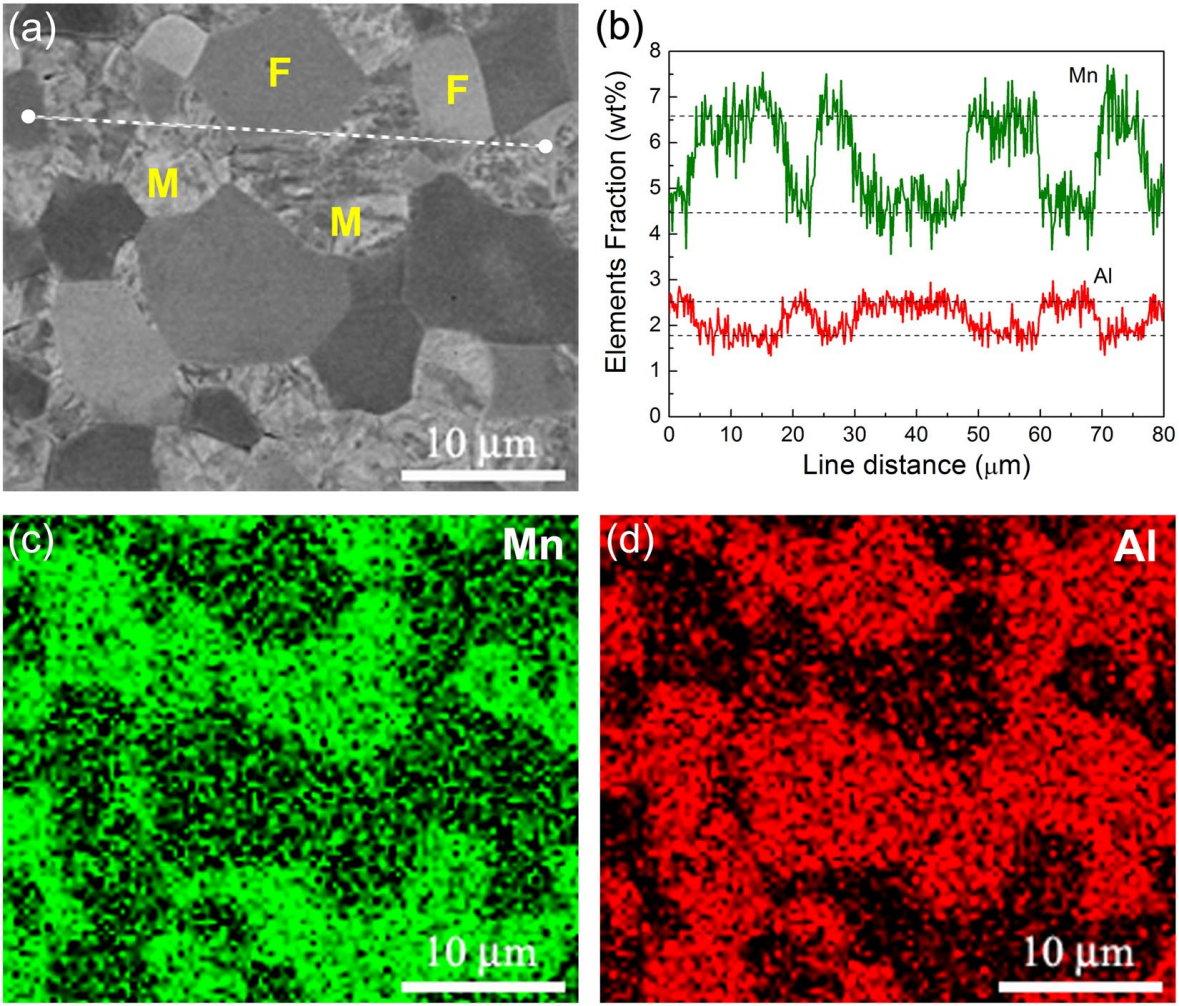
Partitioning of alloying elements between ferrite and martensite in the deformed gauge section of 0.10C5Mn2Al steel after superplastic deformation at 850 °C and 1 × 10^−2^ s^−1^. (**a**) SEM image. The ferrite and martensite grains are marked with F and M, respectively. (**b**) Element line distribution obtained along the white dot line in (**a**). (**c**) Mn distribution. (**d**) Al distribution.

The reverse phase transformation from ferrite to austenite at elevated temperature is controlled by uphill diffusion of solid solution elements, Mn into austenite and Al into ferrite, respectively. Partitioning of each element between austenite and ferrite is shown in Fig. 3. Clearly, Mn is enriched in martensite (austenite) while the Al content is higher in ferrite. An important thing is that the diffusion coefficients of Mn and Al in austenite and ferrite are much lower than that of carbon (Extended Data Fig. 4a), which enhances the stability of both phases significantly though plastic deformation might improve the diffusion of Mn and Al elements. Comparing Fig. 1d and Extended Data Fig. 4b suggests that the highest superplasticity corresponds to austenite volume fraction of 50–60%, which is consistent with the measured value of about 56 ± 4%. It is possible that at this phase ratio the austenite and ferrite grains distributed more randomly and uniformly (Fig. 2d and e), where the neighboring grains in different phase can inhibit rapid grain growth each other by hindering boundary migration, due to the slow uphill diffusion of Mn and Al. Therefore, austenite reverse phase transformation produces large amount of fine grains while sluggish uphill partitioning of solid solution elements maintains grains in a reasonable small size scale. A limited grain growth to a certain extend (e.g. 5–8 μm in Fig. 2c and d) during deformation is allowed in the studied steel, which yield another important effect, strain hardening. It is understood that strain hardening during superplastic deformation at constant strain rate and temperature is attributed to grain growth, which increases the flow stress in accordance with the grain size dependence^18, 19^. On the other hand, the reverse phase transformation from ferrite to austenite can also contribute to strain hardening, because it is well known that the strength of austenite at high temperature is higher than that of ferrite. The volume fraction of austenite increases gradually with increasing time and tensile strain, which enhances the strain hardening as shown in Fig. 1c and Extended Data Figs 1 and 2.

In addition to small grain size, strain rate sensitivity (*m*) favors superplasticity significantly when it is high (typically above 0.3), because it renders materials high resistance to necking. By means of strain rate jump test (Extended Data Fig. 5a and b), it was measured that the *m* value of 0.10C5Mn2Al steel at the beginning of deformation was as high as 0.5 and decreased slowly with increasing strain and strain rate (Extended Data Fig. 5c–f). The critical *m* value of 0.3 can be satisfied even at high strain rate of 10^−2^ s^−1^ and at large engineering strain of about 5.6. The largest superplasticity (ΔL/L_0_ > 1100%) is obtained at about 800 °C and at strain rate of 10^−3^ s^−1^, which is related to *m* value of 0.38–0.52.

Our experimental results show that dynamic austenite reverse phase transformation plays a crucial role for the generation of sliding boundaries (both GBs and phase boundaries). The formation mechanism of high-angle GBs is completely different from those mechanisms by grain nucleation and growth^20^ or by misorientation accumulation through severe plastic deformation^21^. Also the fine grain size could be retained by limited grain growth that is controlled by sluggish uphill partitioning of Mn and Al, which is also different from the rapid grain growth behavior controlled by the fast diffusion of carbon element in conventional low carbon low alloy steels^8, 12^ and the carbide pinning of grain growth in ultrahigh carbon steels^8, 9^. Both these two features are related to our novel chemical composition design of the new low carbon low alloy steels.

**Figure 4:**
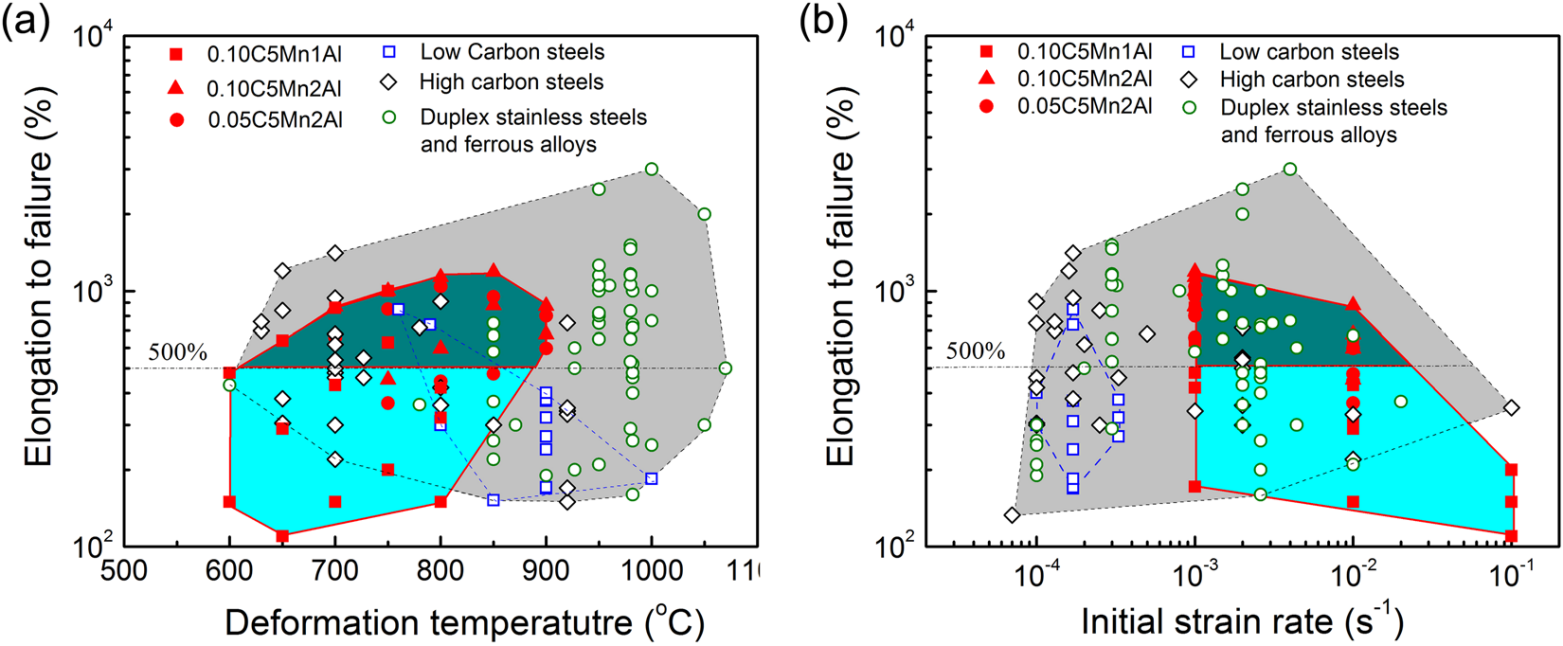
Comparison of superplasticity between current steels and other ferrous alloys reported in literature: (**a**) elongation-to-fracture as a function of deformation temperature and (**b**) elongation-to-fracture as a function of initial deformation strain rate. The referenced data from literature include low carbon steels^8, 12^, high and ultrahigh carbon steels^8, 9, 11, 22–25^, duplex stainless steels and ferrous alloys^8, 10, 26–28^.

The superplasicity of the studied low carbon low alloyed steels can be compared with other typical superplastic ferrous alloys. Figure 4a and b present the plots of superplasticity against deformation temperature and strain rate, respectively, based on the review paper by Maehara *et al* ^8^. and some typical publications in recent two decades^9–12, 22–28^. The dark shaded area highlights those data of the studied steels that can satisfy the industrial requirement (ΔL/L_0_ > 500%) for superplastic forming. In comparison with the high temperature (around 1000 °C) at which the optimum superplasticity of duplex stainless steels can be obtained, the studied steels show much lower deformation temperature of about 800 °C even at a similar strain rate of about 10^−3^ s^−1^, as seen in Fig. 4. Compared with high-carbon steels, the strain rate at which the superplasticity can meet industrial requirement is 1–2 order magnitude higher in the studied steels. Such excellent superplasticity at both high strain rate and relatively low temperature provides an opportunity for current low carbon low alloy steels to fabricate complex components by rapid superplastic forming.

In summary, excellent superplasticity has been achieved in our newly designed low carbon steels produced by simple and commonly applied rolling techniques in steel plants. The excellent superplasticity is attributed to sliding GBs formation by the dynamic austenite reverse phase transformation, and the limited grain growth controlled by sluggish element uphill partitioning. The demonstrated results, not only challenges the traditional understanding that low carbon steel cannot offer suerplasticity, but also offers a practical approach to explore the commercial application of low carbon steel as one of the superplastic materials with low cost and high strength in automobile, aeronautical and astronautical industry.

## Methods

### Material Fabrication

The chemical composition of newly designed steels with C of 0.05–0.15%, Mn of 5% and Al of 0–2% are given in Extended data Table 1, in which both Mn and Al are added to refine microstructure and retard grain growth. The steels were prepared by high frequency induction furnace in a vacuum atmosphere and casted into ingots with weight of 50 Kg. Then the ingots were heated up to 1200 °C with 2 hours and then hot forged into slabs with dimension of 40 × 100 × 200 mm^3^. Thereafter, the slabs were reheated to 1200 °C for 2 hours and then hot rolled into steel sheets with a thickness of 6 mm at final rolling temperature of above 900 °C and air cooled to RT. Finally, the hot rolled steel sheets were further coldly rolled to a final thickness of 1.8 mm at RT.

### Mechanical Testing

Uniaxial tensile tests were conducted on MTS880 tensile machine equipped with temperature controlled chamber. The deformation temperature was controlled within ± 2 degrees. The thin sheet tensile specimens with gauge length of 5 mm, width of 8 mm and thickness of 1.8 mm were cut by electronic line from the cold rolled sheets with tensile direction parallel to the rolling direction. The uniaxial tensile tests and strain rate jump tests were performed at different temperatures of 600 °C, 650 °C,700 °C, 750 °C, 800 °C, 850 °C and 900 °C with different initial strain rates of 10 s^−1^, 10^−2^ s^−1^ and 10^−3^ s^−1^. The superplastic elongation was calculated by *(L-L0)/L0*, where *L0* and *L* are the total length of the gauge length before and after tensile deformation.

### Microstructure Characterizations

The LEO1530 scanning electron microscopy (SEM) equipped with electron-backscattered diffraction (EBSD) and the HITACHI H-800 transmission electron microscopy (TEM) of were employed for microstructural characterizations. ZEISS Gemini Sigma 300 equipped in LEO1530 SEM was applied for the measurement of element distribution of Mn and Al by line scanning and area scanning. The raw EBSD data was processed by orientation averaging using the VMAP software to reduce orientation noise. Further analysis of these EBSD data was carried out by HKL software (Channel 5). The volume fractions of the phases were calculated by using the Thermo-Calc software with TCFE 7 database provided by the CISRI-TCS Joint Open Laboratory.

## Electronic supplementary material

Supplementary materials

## References

[1] Rosenhain W, Haughton JL, Bingham KE (1920). Zinc alloys with aluminum and copper. J. Inst. Met..

[2] Chokshi AH, Mukherjee AK, Langdon TG (1993). Superplasticity in advanced materials. Mater. Sci. Eng. R..

[3] Boyer RR (1996). An overview on the use of titanium in the aerospace industry. Mater. Sci. Eng. A.

[4] Alabort E, Putman D, Reed RC (2015). Superplasticity in Ti-6Al-4V: Characterisation, modeling and applications. Acta Mater..

[5] Mishra RS, Bieler TR, Mukherjee AK (1995). Superplasticity in powder metallurgy aluminum alloys and composites. Acta Metal. Mater..

[6] Ma ZY, Mishra RS, Mahoney MW (2003). Superplastic deformation behavior of friction stir processed 7075Al alloy. Acta Mater..

[7] Kubota K, Mabuchi M, Higashi K (1999). Processing and mechanical properties of fine-grained magnesium alloys. J. Mater. Sci..

[8] Maehara Y, Langdon TG (1990). Superplasticity of Steels and Ferrous Alloys. Mater. Sci. Eng. A.

[9] Zhang H, Bai B, Raabe D (2011). Superplastic martensitic Mn-Si-Cr-C steel with 900% elongation. Acta Mater..

[10] Sagradi M, Pulino-Sagradi D, Medrano RE (1998). The effect of the microstructure on the superplaticity of a duplex stainless steel. Acta Mater..

[11] Walser B, Sherby OD (1975). Mechanical-behavior of superplastic ultrahigh carbon-steels at elevated-temperature. Metall. Trans. A.

[12] Morrison WB (1968). Superplasticity of low alloy steels. Trans. ASM.

[13] Barnes AJ (2007). Super-plasticity forming 40 years and still growing. J. Mater. Sci. Performance.

[14] Cao WQ (2012). Microstructures and mechanical properties of the third generation automobile steels fabricated by ART-annealing. Science China.

[15] Shi J (2010). Enhanced work-hardening behavior and mechanical properties in ultrafine-grained steels with large-fractioned metastable austenite. Scr. Mater..

[16] Jia D (2001). Deformation behavior and plastic instabilities of ultrafine-grained titanium. Appl. Phy. Lett..

[17] Perevezentsev VN, Rybin VV, Chuvildeev VN (1992). The theory of structural superplasticity-III. Boundary migration ad grain-growth. Acta Metall. Mater..

[18] McFadden SX, Mishra RS, Valiev RZ, Zhilyaev AP, Mukherjee AK (1999). Nature..

[19] Perevezentsev VN, Rybin VV, Chuvildeev VN (1992). The theory of structural superplasticity-I. The physical nature of superplasticity phenomenon. Acta Metall. Mater..

[20] Humphreys FJ (1997). A unified theory of recovery, recrystallization and grain growth, based on the stability and growth of cellular microstrcutures-I. the basic model. Acta Mater..

[21] Hughes DA, Hansen N (1997). High angle boundaries formation by grain subdivision mechanisms. Acta Mater..

[22] Talefl EM, Nagao M, Higashi K, Sherby OD (1996). High-strain-rate superplasticity in ultrahigh-carbon steel containing 10wt.%Al (UHCS-10Al). Scr. Mater..

[23] Zhang H (2014). Enhanced superplasticity in an Al-alloyed multicomponent Mn-Si-Cr-C steel. Acta Mater..

[24] Moshksar MM, Rad EM (1998). Effect of temperature and strain rate on the superplastic behavior of high-carbon steel. J. Mater. Proc. Tech..

[25] Furuhara T, Sato E, Mizoguchi T, Furimoto S, Maki T (2002). Grain boundary character and superplasticity of fine-grain ultra-high carbon steel. Mater. Trans..

[26] Sagradi M, Pulino-sagradi D, Medrano RE (1998). The effect of the microstructure on the superplasticity of a duplex stainless steel. Acta Mater..

[27] Li S, Ren X, Li X, Gui Y (2014). Effects of microstructure changes on the superplasticity of 2205 duplex stainless steel. Mater. Des..

[28] Han YS, Hong SH (1999). Microstructral changes during superplastic deformation of Fe-24Cr-7Ni-3Mo-0.14N duplex stainless steel. Mater. Sci. Eng. A.

