# Supplementary material for: Dynamic reverse phase transformation induced high-strain-rate superplasticity in low carbon low alloy steels with commercial potential
Source: Sci Rep. 2017 Aug 23;7:9199. doi: 10.1038/s41598-017-09493-7 (PMC5569066; doi:10.1038/s41598-017-09493-7)

Supplementary materials

Dynamic reverse phase transformation induced high-strain-rate superplasticity in low carbon low alloy steels with commercial potential

Wenquan Cao[1], Chongxiang Huang[2], Chang Wang[1], Han Dong[1] & Yuqing Weng[1]

1. Special Steel department of Central Iron and Steel Research Institute (CISRI), Beijing 100081, China.
2. School of Aeronautics and Astronautics, Sichuan University, Chengdu 610065, China

Extended Data Table 1 Chemical composition of the studied steels

Steels	Chemical composition, weight%			
	Fe	C	Mn	Al
0.10C5Mn	Bal.	0.11	5.2	0.02
0.10C5Mn1Al	Bal.	0.09	4.7	0.8
0.10C5Mn2Al	Bal.	0.10	4.9	2.1
0.05C5Mn2Al	Bal.	0.06	4.8	1.9

Extended Data Figure 1 Tensile stress-strain curves of 0.10C5Mn1Al steel at different initial strain rates and temperatures: (a-b) 1×10^{-3} s^{-1}, (c-d) 1×10^{-2} s^{-1}, (e-f) 1×10^{-1} s^{-1}.

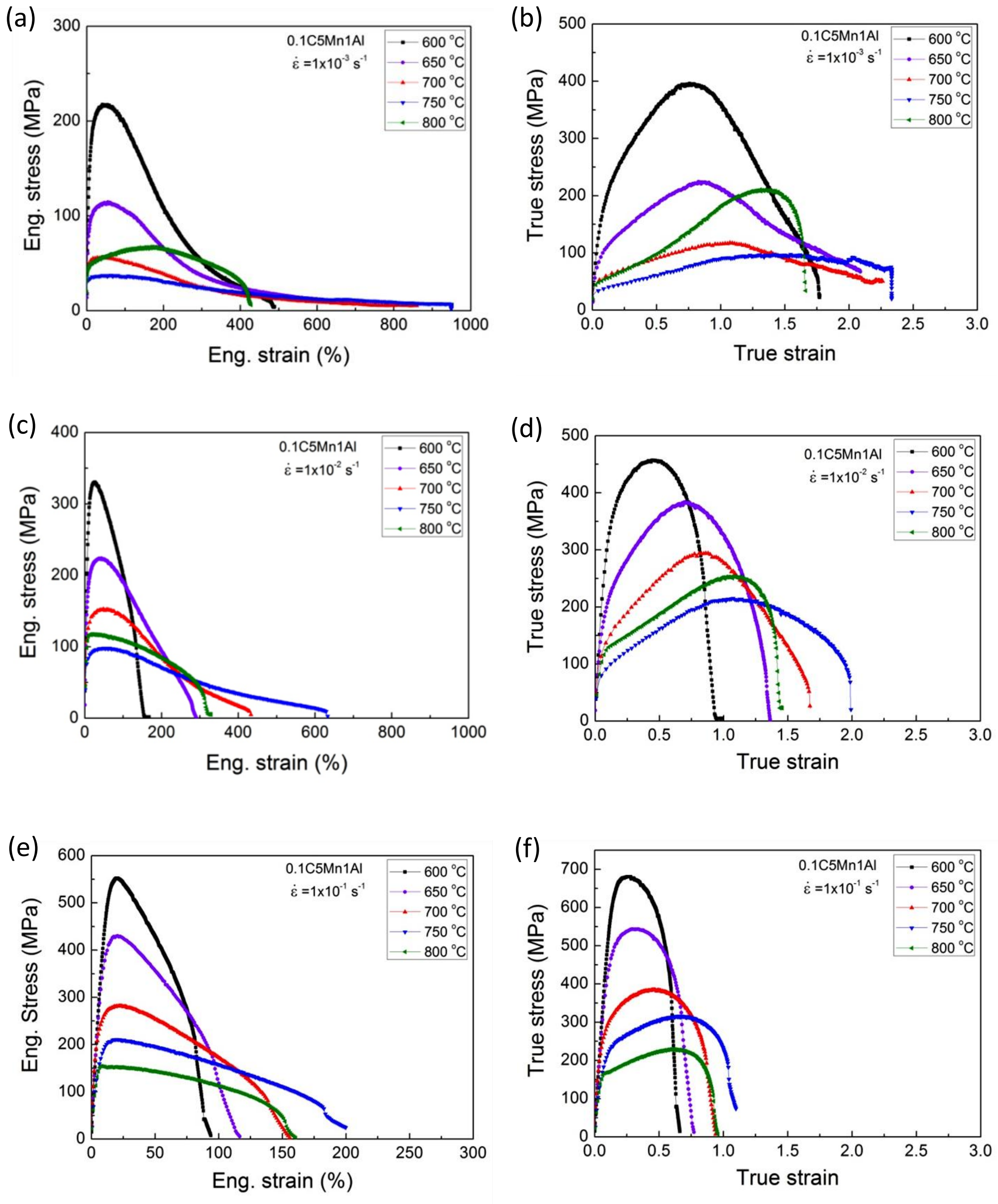

Extended Data Figure 2 Tensile stress-strain curves of 0.05C5Mn2Al steel at different initial strain rates and temperatures: (a-b) 1×10^{-3} s^{-1}, (c-d) 1×10^{-2} s^{-1}.

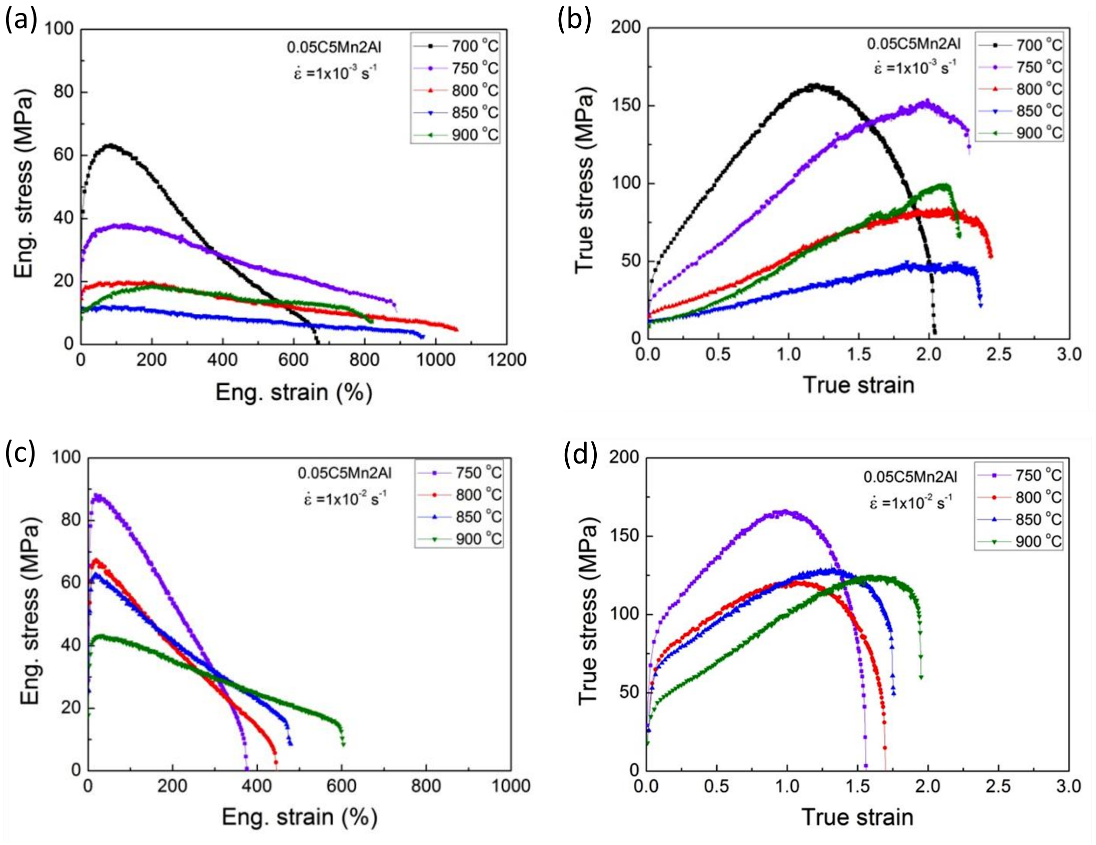

Extended Data Figure 3 Tensile samples before and after deformation: (a) 0.10C5Mn1Al, (b) 0.10C5Mn2Al, (c) 0.05C5Mn2Al.

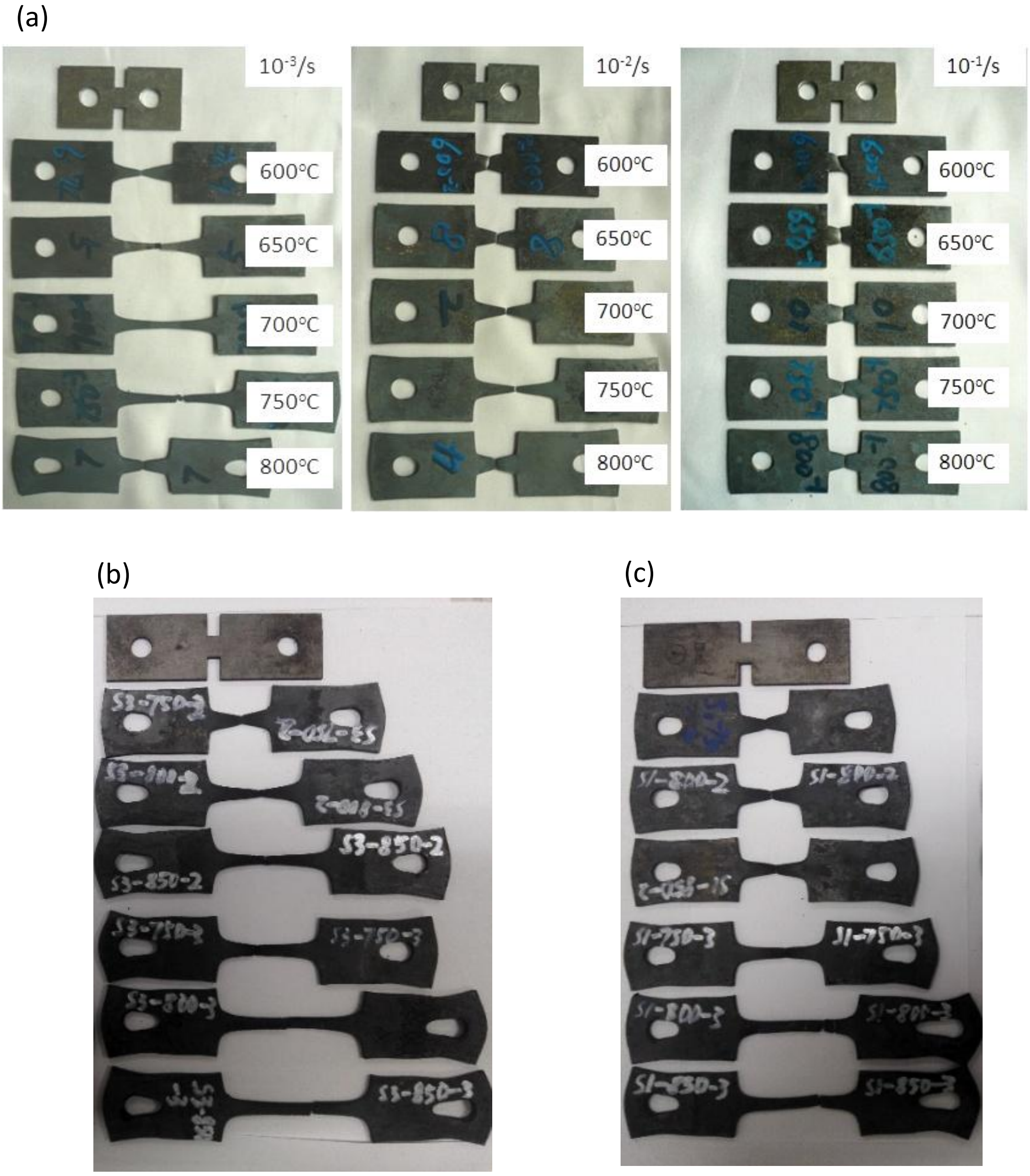

Extended Data Figure 4 (a) Comparison of the diffusion coefficient of C, Mn and Al in α (bcc) and γ (fcc) phases in steel at given temperature range. (Data from: Brandes, E. A. Smithells metals reference book. 6 edition, london: butterworths, 1983). (b) Phase fraction of austenite and ferrite at high temperature in the studied steels by using the Thermo-Calc software with TCFE 7 database.

(a)

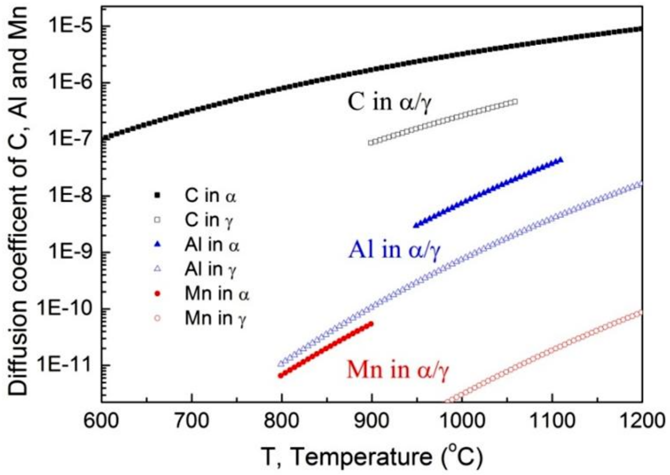

(b)

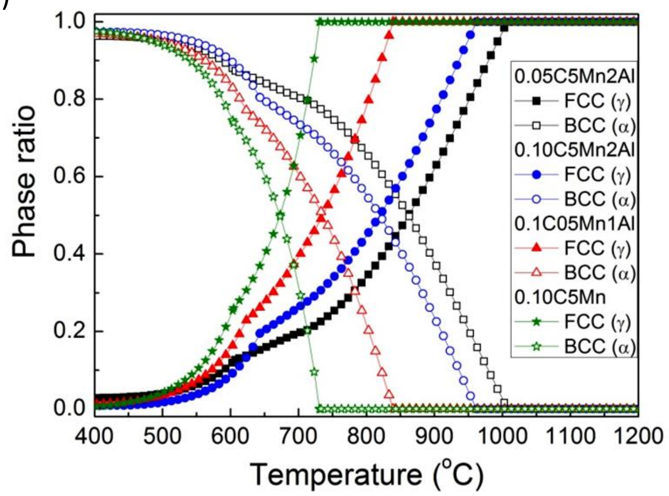

Extended Data Figure 5 (a) Strain rate jump test of 0.10C5Mn2Al steel deformed at different temperatures. (b) Enlarge curves tested at 900 ^{o}C. (c) Plots of *m* values vs. strain at 700 ^{o}C. (d) Plots of *m* values vs. strain at 800 ^{o}C. (e) Plots of *m* values vs. strain at 900 ^{o}C. (f) The calculated *m* values as a function of strain rate.

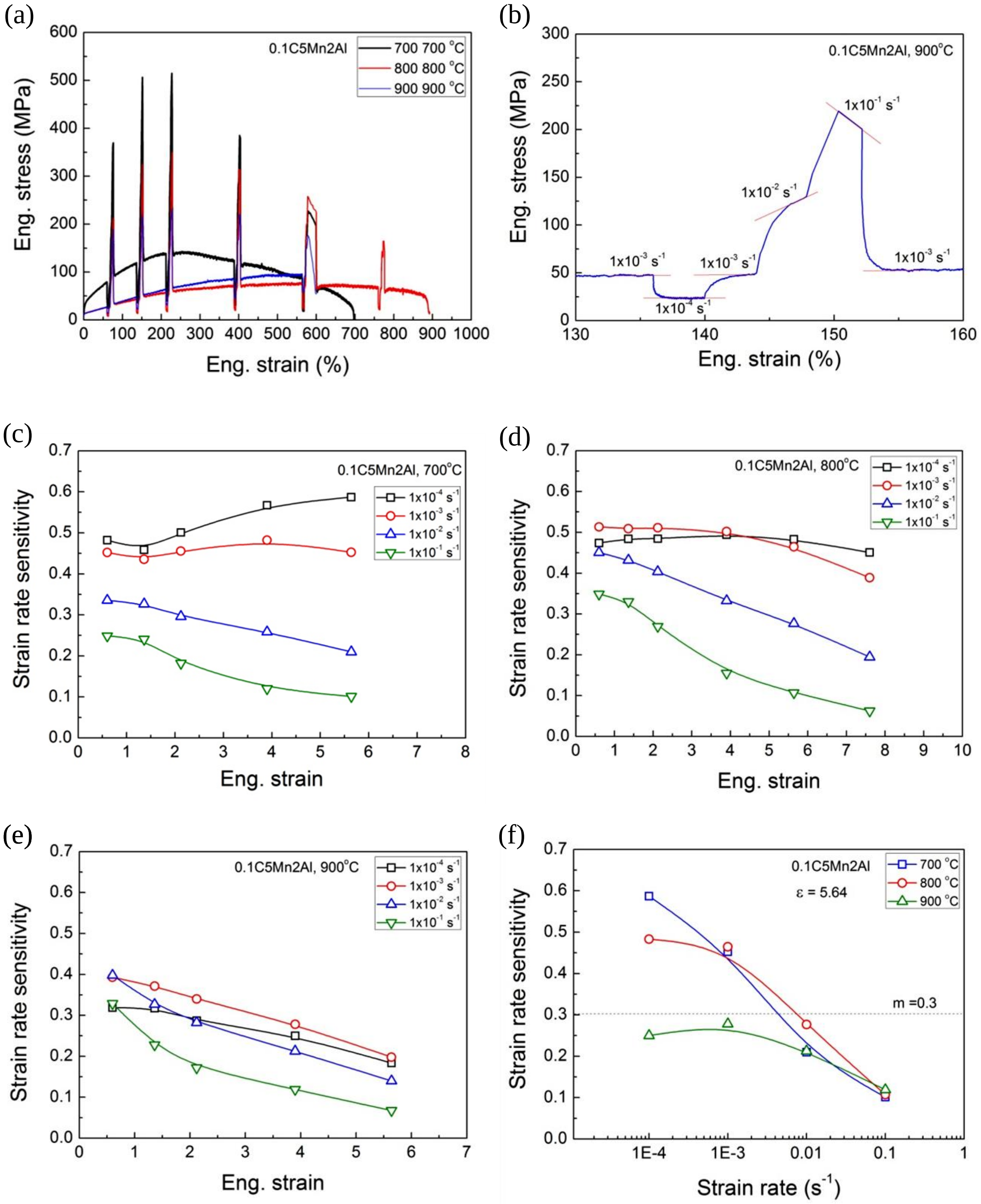

Supplement: Supplementary file 1 — Supplementary materials [file 41598_2017_9493_MOESM1_ESM.pdf]
